# Differential trafficking of AMPA receptors following activation of NMDA receptors and mGluRs

**DOI:** 10.1186/1756-6606-4-30

**Published:** 2011-07-27

**Authors:** Thomas M Sanderson, Graham L Collingridge, Stephen M Fitzjohn

**Affiliations:** 1 MRC Centre for Synaptic Plasticity, School of Physiology and Pharmacology, University of Bristol, Bristol, BS8 1TD, UK; 2 Department of Brain & Cognitive Sciences, College of Natural Sciences, Seoul National University, Gwanak-gu, Seoul 151-746, South Korea; 3 Eli Lilly and Co, Erl Wood Manor, Windlesham, GU20 6PH, UK

**Keywords:** Synaptic plasticity, long-term depression, DHPG, GluA2, NMDA, mGluR, super ecliptic phluorin

## Abstract

The removal of AMPA receptors from synapses is a major component of long-term depression (LTD). How this occurs, however, is still only partially understood. To investigate the trafficking of AMPA receptors in real-time we previously tagged the GluA2 subunit of AMPA receptors with ecliptic pHluorin and studied the effects of NMDA receptor activation. In the present study we have compared the effect of NMDA receptor and group I mGluR activation, using GluA2 tagged with super ecliptic pHluorin (SEP-GluA2) expressed in cultured hippocampal neurons. Surprisingly, agonists of the two receptors, which are both able to induce chemical forms of LTD, had clearly distinct effects on AMPA receptor trafficking. In agreement with our previous work we found that transient NMDA receptor activation results in an initial decrease in surface GluA2 from extrasynaptic sites followed by a delayed reduction in GluA2 from puncta (putative synapses). In contrast, transient activation of group I mGluRs, using DHPG, led to a pronounced but more delayed decrease in GluA2 from the dendritic shafts. Surprisingly, there was no average change in the fluorescence of the puncta. Examination of fluorescence at individual puncta, however, indicated that alterations did take place, with some puncta showing an increase and others a decrease in fluorescence. The effects of DHPG were, like DHPG-induced LTD, prevented by treatment with a protein tyrosine phosphatase (PTP) inhibitor. The electrophysiological correlate of the effects of DHPG in the SEP-GluA2 infected cultures was a reduction in mEPSC frequency with no change in amplitude. The implications of these findings for the initial mechanisms of expression of both NMDA receptor- and mGluR-induced LTD are discussed.

## Background

AMPA receptor trafficking is under exquisite control in excitatory neurons (reviewed in [1,2]). One way to change the efficacy of a synapse is to redistribute AMPA receptors at the postsynaptic membrane so as to either increase or decrease their number and thus alter the responsiveness of the synapse to glutamate. Such changes in synaptic efficacy, termed synaptic plasticity, are crucial for normal brain function, particularly during the development of synaptic connections and memory formation. One form of plasticity, long term depression (LTD), involves a decrease in synaptic strength and can occur via trafficking of AMPA receptors away from synapses. Two major forms of LTD have been described in the CNS that are triggered by the activation of NMDA and mGluRs. These are induced physiologically by trains of electrical stimulation [3-5] but can also be mimicked by the application of specific agonists, in particular N-methyl D-aspartate (NMDA) [6,7] and dihydroxyphenylglycine DHPG [8-10], respectively.

For NMDA-induced LTD there is agreement between electrophysiological and imaging studies on the importance of AMPA receptor endocytosis in LTD expression [1,3,11,12]. In the case of mGluR-induced LTD (mGluR-LTD), however, conflicting evidence has been reported [13]. Immunofluorescence and biochemical studies indicate that surface AMPA receptor numbers decrease on exposure to DHPG [14-16]. However, a range of electrophysiological measurements, such as changes in paired-pulse facilitation [14,17-20], failure rate [17], coefficient of variation [14,17] and mEPSC parameters [14,17], are more indicative of a presynaptic locus of expression. Consistent with this, recordings from adult hippocampal slices show no change in postsynaptic sensitivity to glutamate following DHPG-induced LTD [21] and in both adult and juvenile hippocampal slices the amount of stimulus-induced zinc exocytosis (a measure of neurotransmitter release) decreases as a result of DHPG-induced LTD [22]. A developmental switch has been suggested as an explanation for these conflicting results with DHPG-induced LTD requiring AMPA receptor redistribution in adolescent rats, but not earlier in ontogeny [23], although changes in both paired-pulse facilitation and the coefficient of variation of EPSC amplitude have been observed as a result of DHPG-induced LTD in adult animals [14], arguing against this hypothesis. Thus the precise role of AMPA receptor trafficking in mGluR-LTD remains unclear.

In the current study we have investigated the role of GluA2-containing AMPA receptor trafficking in DHPG-induced LTD. We have used a version of GluA2 tagged with super ecliptic pHluorin (SEP-GluA2; [24]). SEP is a variant of green fluorescent protein engineered so that its fluorescence is eclipsed when in acidic environments [25]. The fluorophore of SEP has a pKa of ~7.1, such that it fluoresces ~20 times more at pH 7.4 than at pH 5.6 [26]. As the lumen of endocytic vesicles is acidic (pH 5.6) [25] SEP fluorescence is eclipsed when in this environment, whereas when in the extracellular environment, which has a pH of 7.4, it is revealed. When SEP is expressed in neurons, the fluorescence under basal conditions is therefore predominantly from surface-expressed SEP.

Here we firstly confirm our previous observations [24] that NMDA results in a rapid and reversible decrease in fluorescence in dendritic shafts and a delayed decrease in fluorescence in puncta (putative spines). Secondly we report that DHPG has very different effects on SEP-GluA2 at both dendritic shafts and puncta. DHPG causes a substantial decrease in SEP-GluA2 in dendritic shafts that progressively develops over a period of 20-30 min and is not reversible during the time-course of these experiments. Even more surprisingly, however, there was no change in the average fluorescence of puncta over this period. We observed both increases and decreases in fluorescence at individual puncta, that we hypothesize may be due to an increase in receptor mobility. The effects of DHPG were prevented by orthovanadate (Ov), a treatment that prevents DHPG-induced LTD [27]. These observations demonstrate that stimulation of two classes of glutamate receptor (NMDA and mGluR) have strikingly different effects on AMPA receptor trafficking.

## Results

### SEP-GluA2 imaging

**Figure 1:**
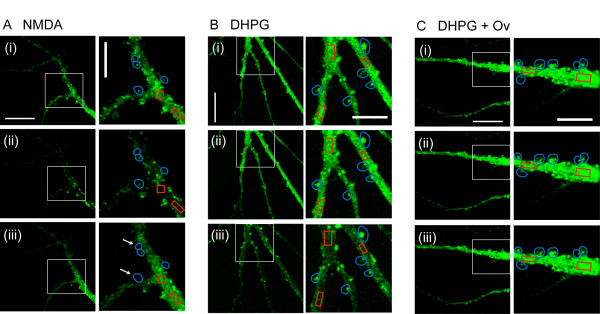
**NMDA and DHPG differentially regulate trafficking of SEP-GluA2 in cultured hippocampal neurons**. A. Representative images of cultured hippocampal neurons infected with SEP-GluA2 prior to NMDA treatment (i), 5 min post addition of NMDA (ii) and 35 min post addition of NMDA (iii). B Representative images of cultured hippocampal neurons infected with SEP-GluA2 prior to DHPG treatment (i), 5 min post addition of DHPG (ii) and 35 min post addition of DHPG (iii). C. Representative images of cultured hippocampal neurons pre treated with 1 mM orthovanadate prior to DHPG treatment (i), 5 min post addition of DHPG (ii), and 35 min post addition of DHPG (iii). Example regions of interest (ROI) of the kind that were used for analysis are highlighted in red (dendritic shaft) and blue (puncta). Calibration represents 20 μm on the main images and 10 μm on insets.

**Figure 2:**
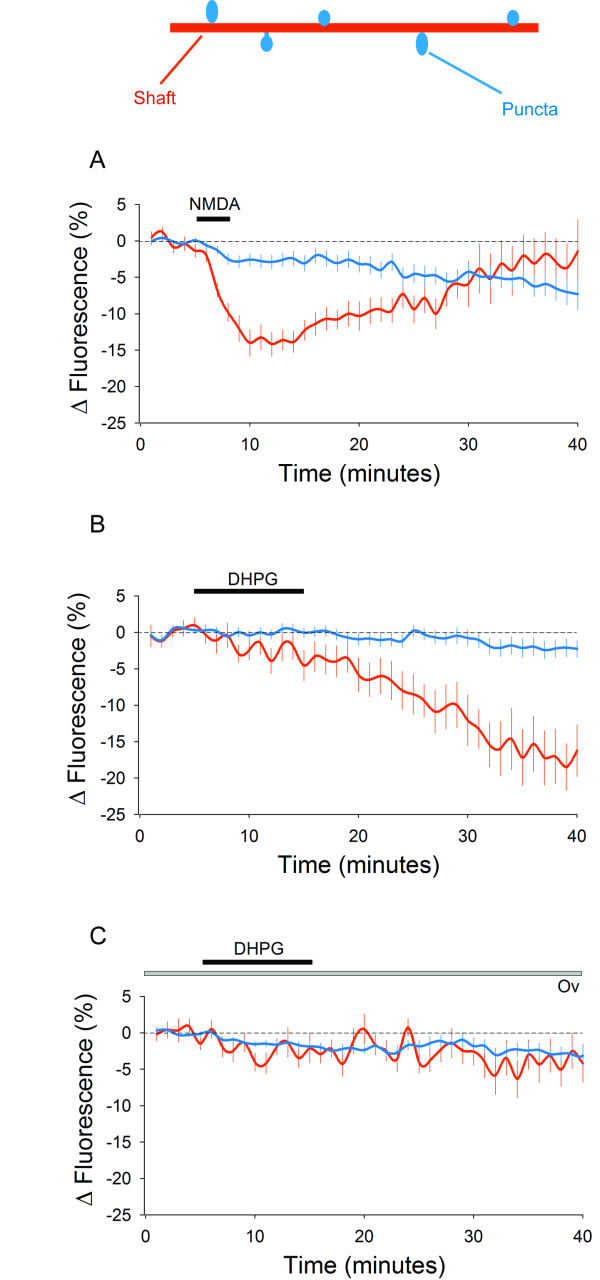
**Analysis of SEP-GluA2 fluorescence in ROIs in the dendritic shaft (red lines) and in punta (blue lines)**. A. Application of NMDA (black bar) results in a rapid and reversible reduction in average fluorescence in the dendritic shaft, followed by a later reduction in average fluorescence in puncta. B. Application of DHPG (black bar) results in a slow reduction in average dendritic shaft fluorescence but no change in average puncta fluorescence over the time period studied. C. The PTP inhibitor Ov applied continuously during the experiment (grey bar) prevents the reduction in shaft SEP-GluA2 fluorescence induced by DHPG (black bar).

**Figure 3:**
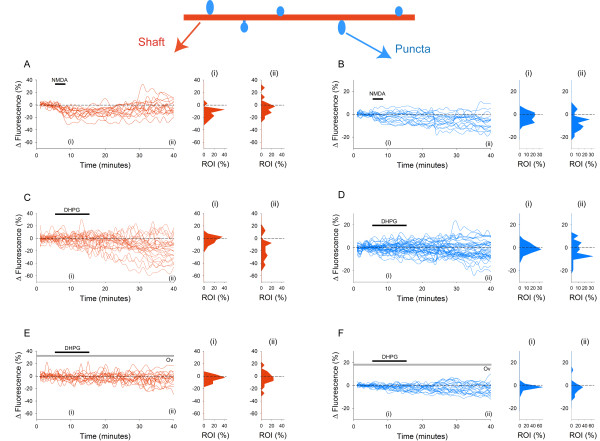
**Analysis of fluorescence from individual ROIs reveals heterogeneous responses to application of agonists**. A. Application of NMDA (black bar) results in a rapid and reversible decrease in fluorescence in ROIs on the dendritic shaft; reflected in the majority of ROIs showing negative values at 10 min (i) but not at 40 mins (ii). B. Application of NMDA (black bar) results in a slow decrease in fluorescence in ROIs on puncta, reflected in many ROIs showing no change in fluorescence at 10 min (i), but decreases in fluorescence at 40 min (ii). C. Application of DHPG (black bar) results in slow decreases in fluorescence in ROIs on the dendritic shaft; reflected in the majority of ROIs showing no change at 10 min (i) but decreased fluorescence at 40 min (ii). D. Application of DHPG (black bar) results in variable changes in fluorescence in ROIs on puncta. Most ROIs show no change in fluorescence at 10 min (i), however at 40 min puncta show both decreases and increases in fluorescence (ii). DHPG applied in the presence of Ov results in a substantially smaller change in fluorescence in ROIs on either the dendritic shaft (E) or puncta (F) at 10 min (i) or at 40 min (ii).

We firstly determined experimental conditions in which photo-bleaching did not cause fluorescence levels to deteriorate significantly over the time period studied. Making image stacks at 1 min intervals for 40 min resulted in only a small decrease in fluorescence of 6 ± 4% in areas of dendritic shaft and 3 ± 1% in puncta, values not significantly different to those at the beginning of the experiments (18 dendritic shaft regions and 25 puncta from 8 cells, p > 0.05; data not illustrated). Under these conditions, NMDA (50 μM, 3 min) caused a transient decrease in SEP-GluA2 fluorescence on areas of the dendritic shaft followed by a decrease in fluorescence in puncta (Figure 1), as previously reported [24]. The NMDA induced reduction in shaft fluorescence was detectable within 1 min of the onset of NMDA application and reached a maximum depression of 14 ± 2% at 6 min after the onset of NMDA application (Figure 2A, 18 dendritic regions from 6 cells, p < 0.05). Subsequently there was a small but significant decrease in the mean fluorescence of puncta compared to control neurons (7 ± 2% at 35 min after onset of NMDA application, in 22 spines from 6 cells, p < 0.05). Inspection of individual puncta revealed considerably variability with some spines showing little or no change whereas others showing marked reductions in fluorescence (Figure 3B). Indeed, in some puncta fluorescence decreased to background levels (Figure 1A, arrows).

In contrast to the effects of NMDA, DHPG application (100 μM, 10 min) resulted, on average, in a more slowly developing decrease in SEP-GluA2 fluorescence in areas of the dendritic shaft, which increased progressively over 20-30 min and showed no signs of reversibility over the time-course of the experiments (Figure 1B). At 30 min, the mean fluorescence levels were reduced by 19 ± 3% (Figure 2B, 28 dendritic shaft regions from 10 cells, p < 0.05). Surprisingly, despite large effects in dendritic shafts, DHPG had essentially no effect on the average fluorescence in puncta (Figure 2B). For example, 35 min after the start of the application of DHPG, fluorescence levels in puncta were reduced by 2 ± 1% of baseline, a value not statistically different to control neurons (Figure 2, 40 spines from 10 cells). Once again, however, the average data concealed considerable heterogeneity at the level of individual puncta, with both increases and decreases observed (Figure 3D).

DHPG-induced LTD occurs via a process involving PTPs [27]. To investigate if the dendritic SEP-GluA2 internalization involves similar molecular mechanisms these experiments were also performed on cultures pre-treated with the PTP inhibitor orthovanadate (Ov; 1 mM). Under these conditions, changes in fluorescence in the dendritic shaft were not significantly different to that in untreated cells (Figure 1C). For example, 35 min after DHPG application, mean fluorescence levels were only reduced by 4 ± 3% (17 dendritic shaft regions from 6 cells, p > 0.05), a value that was significantly different to that for dendrites to which DHPG had been applied alone (p < 0.05). Fluorescence in dendritic puncta also did not vary significantly under these conditions. For example, 35 min after DHPG application fluorescence levels in spines were reduced by only 3 ± 1% (Figure 2C, 20 spines from 6 cells, p > 0.05), a value not significantly different from untreated neurons. Analysis of individual events revealed much less variability, compared with the effects of DHPG alone (Figure 3E-F).

### Electrophysiological characterization of DHPG-induced LTD in hippocampal cultures

**Figure 4:**
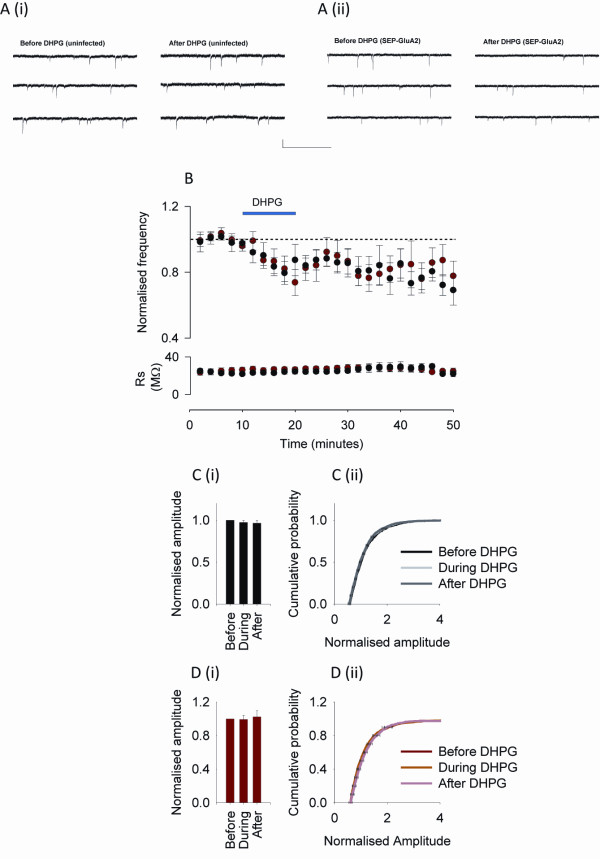
**Analysis of mEPSCs in infected and uninfected cultured hippocampal neurons reveals a selective decrease in mEPSC frequency**. A. Representative examples of traces from (i) uninfected neurons, and (ii) SEP-GluA2 infected neurons. Calibration, 20 pA and 1 s. B. Application of DHPG (blue bar) results in a reduced mEPSC frequency in uninfected (black points) and infected (red points) neurons. C. Analysis of mEPSC amplitudes from uninfected neurons suggests that (i) DHPG does not affect mean amplitudes during or 30 min after DHPG application, and (ii) does not affect the cumulative probability distribution at the same time points. D. Analysis of mEPSC amplitudes from SEP-GluA2 infected neurons suggests that (i) DHPG does not affect mean amplitudes during or 30 min after DHPG application, and (ii) does not affect the cumulative probability distribution at the same time points. Data are presented as mean ± SEM.

We confirmed that DHPG is able to affect synaptic transmission in our preparation by recording miniature excitatory postsynaptic currents (mEPSCs) in SEP-GluA2 infected (n = 8) and uninfected (n = 8) neurons from the same cultures. DHPG (100 μM, 10 min) resulted in a reduction in the frequency of mEPSCs as previously observed [14,17]. The frequency 30 min following DHPG wash out was significantly reduced compared to baseline in both infected and uninfected neurons, at 82 ± 7% and 74 ± 6% of baseline, respectively (Figure 4A-B, p < 0.05). No significant difference was observed in the amplitude of mEPSCs after DHPG application. The mean amplitude 30 min post DHPG application was 96 ± 4% (Figure 4Ci) and 99 ± 5% (Figure 4Di) of baseline in infected and uninfected neurons, respectively (p > 0.05). As the effect of DHPG on mEPSC frequency appears rapidly, we also measured the mEPSC amplitudes during the period of drug application itself. No change in mEPSC amplitude was detected during DHPG application either. The mean amplitudes during the DHPG application were 97 ± 2% and 102 ± 7% of baseline in infected (Figure 4Ci) and uninfected (Figure 4Di) neurons, respectively (p > 0.05). In addition the shapes of the cumulative probability plots in both treatment groups were not significantly different before, during or after DHPG application, suggesting no change in the distribution of amplitudes within the data sets (Figure 4Cii-Dii, p > 0.05).

## Discussion

In the present study we have compared the effects of activation of NMDA receptors and mGluRs on AMPA receptor trafficking, using SEP-GluA2 to enable the dynamics of surface expressed AMPA receptors to be studied in real time. Our primary finding is that there are clear differences in the behaviour of AMPA receptors depending on the class of glutamate receptor that is activated. NMDA causes a rapid but reversible decrease in surface AMPA receptors on dendritic shafts and a delayed decrease in AMPA receptors on the surface of puncta. In contrast, DHPG causes a more slowly developing but persistent decrease in surface AMPA receptors on dendritic shafts but no net change on puncta. Analysis of individual puncta, however, revealed considerable heterogeneity: NMDA stimulation caused mainly either decreases or no change whereas DHPG additionally caused increases in surface AMPA receptors. Overall, these results are consistent with the hypothesis that the effect of DHPG is to mobilise surface AMPA receptors; an effect that is inhibited by treatment with Ov.

### The use of SEP-GluA2 to monitor AMPA receptor trafficking

We have focussed here on GluA2 as levels of phosphorylation and surface expression of this subunit are altered with the induction of mGluR-LTD [14,28-30]; indeed the presence of the GluA2 subunit is required for mGluR-LTD induction [31]. We have used a pHluorin-tagged GluA2 subunit, which previous work has suggested provides a reliable indicator of native AMPA receptor trafficking [24,32-36]. The majority of SEP-GluA2 puncta colocalise with the synaptic markers synaptophysin and FM4-64 [24], indicating puncta represent GluA2 located at active synapses.

### Implications for the locus of expression of NMDAR-LTD

Previous work using electrophysiological readouts [3,11], immunocytochemistry [3,11] and pHluorin-tagged receptors [24,32] has identified the synaptic removal and internalisation of AMPA receptors as a major component of NMDAR-LTD. Our previous work, using the latter technique, has presented evidence that AMPA receptors are initially internalised from the dendritic shaft and this is followed by a loss of synaptic AMPA receptors and re-population of the dendritic shaft. Based on this we proposed a two-state model comprising internalisation at non-synaptic regions followed by lateral diffusion from synapses to re-populate the extrasynaptic regions and decease synaptic strength. Subsequent work has supported this model and has used the transient decrease in shaft GluA2 as an AMPA receptor internalisation assay. Our current work is also consistent with the two-state model.

The analysis of individual puncta showed marked variability in their behaviour to NMDA receptor activation. In some cases there were no apparent changes in fluorescence and in others the fluorescence decreased such that it was no longer detectable above background. Whether this corresponds to the silencing of synapses, a phenomenon that has been observed electrophysiologically during NMDAR-LTD (Luthi et al, 1999), remains to be established. Clearly, however, there was a wide spectrum of changes, which is most compatible with a model where many synapses lose some, but not all, of their surface expressed AMPA receptors in response to NMDA receptor activation.

### Implications for the locus of expression of mGluR-LTD

The locus of expression of mGluR-LTD is still unresolved. Previous electrophysiological studies on DHPG-induced LTD have shown changes in electrophysiological parameters, such as failure rate and coefficient of variation of EPSC amplitude (reviewed in [13]), which are consistent with presynaptic changes in release probability (Pr) but which may also result from complete loss of AMPA receptors from individual synapses. Changes in paired-pulse facilitation are also observed [14,17-20,28] and are generally believed to reflect changes in Pr [37] but could be explained by the selective silencing of synapses that have high initial Pr (see [17]). In addition, DHPG-induced LTD in adult animals is not associated with changes in photoreleased glutamate [21], which is also consistent with a presynaptic locus of expression, though could be due to a rearrangement of AMPA receptors on the plasma membrane surface. On the other hand, substantial reductions in the number of puncta upon mGluR stimulation have been consistently described in previous studies on cultured hippocampal neurons using antibodies to label native receptors [14,15,29]. Also, decreases in surface AMPA receptors have been measured by non-fluorescence methods [14,28,30].

**Figure 5:**
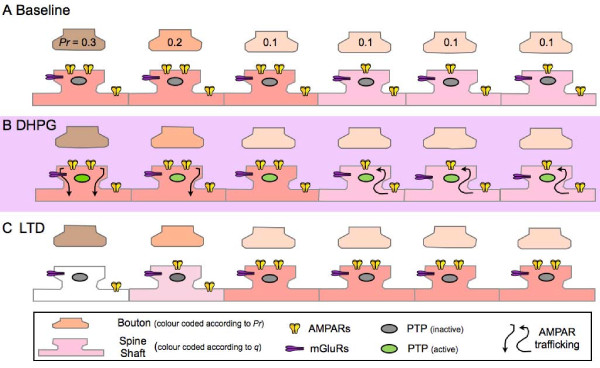
**A potential mechanism for the expression of DHPG-induced LTD, based on the mobilization of AMPA receptors**. A. Baseline: Synapses have a wide distribution of *Pr *skewed in favour of low probabilities of release. In this scheme *Pr *does not alter. There is also a wide range of quantal amplitudes (*q*), which are shown simplified here as giving rise to strong (2 AMPA receptors) and weak (1 AMPA receptor) synapses. B. Induction: Stimulation of mGluRs (mGluR5) by DHPG transiently activates PTPs to dephosphorylate and mobilize AMPA receptors. At high Pr synapses, AMPA receptors are internalized (and degraded) to either result in silencing (in a minority of very high *Pr *synapses) or a decrease in *q*. At a proportion of low Pr synapses AMPA receptors are inserted to maintain a constant level of AMPA receptors available to respond to synaptic release. These could be delivered from extrasynaptic sites by lateral diffusion, thereby leading to the decrease in this AMPA receptor population. C. Expression: In the simplified scheme shown here there would be a large decrease in the mean synaptic current (due to the silencing of a high *Pr*, strong synapse), no change in sensitivity to L-glutamate (localized to synapses by uncaging), no change in mEPSC amplitude or its quantal distribution but a decrease in average *Pr *(due to the functional elimination of a high *Pr *synapse - i.e., a reduction in *n*), a corresponding decrease in mEPSC frequency and an increase in PPF (due to the selective silencing of a high *Pr *synapse).

Is it possible to explain all of these observations with a single model? A mechanism that only involves the postsynaptic neuron could account for these data. This is based on the observation that DHPG, whilst causing no net change, induces both increases and decreases in SEP-GluA2 at individual synapses. Speculatively, there could be internalisation of AMPA receptors at high Pr synapses and a compensatory increase in the surface expression of AMPA receptors at low Pr synapses. Provided that there was complete silencing at some high Pr synapses then this would account for the reported electrophysiological observations. If the neuron maintained a constant level of synaptic AMPA receptors, and also conserved the distribution of AMPA receptors between the complement of functional synapses, then this could explain both the lack of change in mEPSC amplitude and the lack of change in sensitivity to uncaged L-glutamate. It would mean, however, that mechanisms exist to compensate for the loss of high Pr synapses by the insertion of AMPA receptors at low Pr synapses. It is known that neurons can normalise synaptic weights over long periods of time via the process of synaptic scaling [38] and, interestingly, that this might involve mGluRs [39]. The hypothesis proposed here would require a similar type of process occurring very rapidly and one which links this direction of AMPA receptor movement to Pr. This could be achieved if the direction of AMPA receptor movement was determined by the level of their activation by synaptically released L-glutamate. In this respect, there is evidence that mGluR-LTD requires co-activation of mGluRs and AMPA receptors [37]. This mechanism could offer an explanation for the marked reduction in AMPA receptors on dendritic shafts. If the internalised AMPA receptors are targeted for degradation then the AMPA receptors that populate low Pr synapses could arrive by lateral diffusion from surrounding dendritic areas. A tentative scheme that summarises these ideas is presented in Figure 5. Further work will, however, be needed to test this hypothesis more directly and to see whether it can account for mGluR-LTD in slice preparations.

### Differential trafficking of AMPA receptors triggered by NMDA receptors and mGluRs

Irrespective of how mGluR-LTD is expressed, the present results clearly demonstrate that AMPA receptor trafficking in cultured hippocampal neurons is differentially regulated by NMDA receptors and mGluRs. Thus, within hippocampal synapses there can exist two distinct forms of LTD that are triggered by the activation of different classes of glutamate receptor, involve different signalling mechanisms and affect AMPA receptors in different ways. The differential trafficking of AMPA receptors subsequent to stimulation of mGluR *versus *NMDA receptors is consistent with other evidence that the two forms of LTD involve distinct signalling mechanisms ([14,28,30], for review see [13,40]), have differential requirements for protein ubiquitination [41] and actin reorganization [31] and may occur at distinct synapses [42].

## Conclusions

The principal finding of the present study is that stimulation of NMDA receptors and mGluRs results in pronounced differences in AMPA receptor trafficking. Further work will be required to understand the functional consequences of such differential regulation.

## Methods

### Cell culture

Male postnatal day 1-2 wistar rats were sacrificed in accordance with the UK Animals (Scientific Procedures) Act 1986. Brains were immediately removed and kept on ice in Dulbecco's modified eagle medium (DMEM). The hippocampi were removed and cut into transverse slices of approximately 500 μm. Slices were washed 4 times in fresh DMEM twice in Hank's balanced salt solution (HBSS) and incubated in DMEM containing trypsin (5 mg/ml) and DNAse (0.2 mg/ml) for 5-15 mins at 37°C. Mechanical dissociation was performed in HBSS containing DNAse (1 mg/ml), dissociated neurons were pelleted by centrifugation (180 g/10 min) and re-suspended in culture media consisting of Neurobasal A supplemented with glutamine or glutamax (2 mM), 2% B27, 10% horse serum (HS) and gentamycin (2 μg/ml). Cells were plated at a density of ~50,000 per dish onto 22 mm or 24 mm glass cover slips coated with poly-L-lysine (1 mg/ml) and maintained at 37°C in a 95% O_2_, 5% CO_2 _environment. On *in vitro *day 3 culture media was supplemented with uridine (10 μM) and 5-fluoro-2'-deoxyuridine (10 μM) to prevent glial cell proliferation. Culture media was changed twice a week. Neurons were used for experiments 13-17 days after plating.

### Live cell imaging

Hippocampal cultured neurons were infected with SEP tagged GluA2 (Ashby et al., 2004a) using Sindbis virus 16 to 24 hours before experimentation. Images were made using the 63 × water-immersion objective lens (numerical aperture, 1.2) of an inverted Zeiss LSM 510 META confocal laser scanning microscope (Zeiss, Oberkochen, Germany) at 37°C. SEP excitation was achieved using 488 nm radiation from an Argon laser and SEP fluorescence was collected through a FITC filter. The imaging parameters were optimised to minimise bleaching of SEP-GluA2, resulting in maximisation of the length of experiments (i.e. the minimum strength of excitation light was used that resulted in images with adequate signal to noise ratios). Image stacks consisting of 1 μm thick slices were constructed to easily include dendrites under study. Fluorescence was confirmed to be from SEP-GluA2 expressed at the surface by washes with MES buffered saline at pH6, in the presence and absence of NH_4_Cl, applied in order to collapses the pH gradient over cell membranes and reveal intracellular SEP [24].

Image stacks were analysed using Volocity 4 3D imaging software (Improvision Inc, MA, USA). Regions of interest (ROIs) were drawn round areas of diffuse fluorescence in the shaft and in spines that could be easily distinguished from the shaft (and so would not be contaminated by shaft fluorescence). Small movements of dendrites were compensated for by manually moving the regions of interest. Average fluorescence intensity from ROIs around areas of shaft or spines were pooled.

### Electrophysiology

Dissociated cultured hippocampal neurons were maintained at room temperature (22-24°C) and perfused at ~2-3 ml/min with HEPES buffered saline (HBS) comprising (in mM): NaCl (121); KCl (3); CaCl_2 _(2); MgCl_2 _(2); HEPES (25); D-glucose (33); tetrodotoxin citrate (0.5 μM); picrotoxin (100 μM); glycine (1 μM); ~320 mOsm; pH adjusted to 7.4 with NaOH. Voltage-clamp whole-cell recordings were made using an Axopatch 200B amplifier (Molecular Devices, Union city, CA, USA). Patch electrodes (~3-5 MΩ) contained whole-cell solution comprising (in mM): caesium-methane sulphonate (135); HEPES (10); EGTA (0.5); NaCl (8); Mg-ATP (4); Na_2_-GTP (0.3); ~290 mOsm; pH adjusted to 7.2 using CsOH. Initial offset potentials were corrected for before recording. Junction potentials were not adjusted for. Continuous recordings sampled at 20 kHz were made using WinLTP [43] and series resistance was measured at 30 s intervals throughout.

mEPSCs were analyzed offline using MiniAnalysis (Synaptosoft Inc., Decatur, GA, USA). Events were detected by setting the threshold value for detection at three times the level of the root mean square noise, followed by visual confirmation of mEPSC detection. Frequency plots were produced by ordering the times that the mEPSCs occurred into 2 min bins. These data were normalised to the 10 min period prior to DHPG application. For construction of cumulative probability plots, 400 successive events were used from the period immediately preceding application of DHPG, from the period during DHPG application, and from the period 25 - 30 min after commencing washout of DHPG. For pooled cumulative probability plots, data were normalized to the median value in the pre-DHPG period.

### Statistical analysis

Statistical significance was determined using Student's t-tests (2 conditions) and using one way analysis of variance (ANOVA) followed by post hoc Bonferroni comparisons (> 2 conditions) in GraphPad Prism v5 (GraphPad Software, Inc., La Jolla, CA, USA). In imaging experiments, significance was assessed by comparing all four conditions using this test, at the time points quoted, in order that the effect of bleaching was taken into account. In order to test for differences in the distribution of mEPSC amplitudes Kolmogorov-Smirnov tests were applied using MiniAnalysis (Synaptosoft Inc., Decatur, GA, USA). The level of significance was set at p < 0.05.

### Drugs

DNAse, D-glucose, 5-fluoro-2'-deoxyuridine, gentamycin, glycine, HEPES, poly-l-lysine, potassium chloride, trypsin and uridine were obtained from Sigma Aldrich (Pool, UK). B27, DMEM, glutamine, glutamax, HBSS, horse serum, Neurobasal A were obtained from Invitrogen (Paisley, UK). Calcium chloride and magnesium chloride were obtained from VWR International Ltd (Poole, UK). (RS)-3,5-dihydroxyphenylglycine (DHPG), picrotoxin, sodium orthovanadate and tetrodotoxin citrate were obtained from Tocris (Bristol, UK).

## Competing interests

The authors declare that they have no competing interests.

## Authors' contributions

TMC designed and performed the experiments, analysed the data and wrote the first draft of the paper.

SMF and GLC conceived and supervised the study, contributed to the design of the experiments and helped write the manuscript.

All authors read and approved the final manuscript.
